# Implementation strategies for embedding patient-reported outcome and experience measures (PROMs/PREMs) in routine care: secondary analysis of an umbrella review

**DOI:** 10.1186/s41687-026-01006-3

**Published:** 2026-01-29

**Authors:** Guillaume Fontaine, Joshua Ramos, Meagan Mooney, Marie-Eve Perron, Laura Crump, Sylvie D. Lambert

**Affiliations:** 1 McGill University, 680 Rue Sherbrooke O # 1800, Montréal, QC H3A 2M7 Canada; 2 Lady Davis Institute for Medical Research, 3755 Chem. de la Côte-Sainte-Catherine, Montréal, QC H3T 1E2 Canada; 3 Ottawa Hospital Research Institute, 501 Smyth Road, Ottawa, ON K1H 8L6 Canada; 4 Kirby Institute, UNSW Sydney Institution, Cnr High St & Botany St, Kensington, NSW 2033 Australia; 5 St. Mary’s Research Centre, 3777 Jean Brillant St, Montreal, QC H3T 0A2 Canada; 6 Research Institute of the McGill University Health Centre Research, 1001 boul. Décarie, Montreal, QC H4A 3J1 Canada

## Abstract

**Background:**

Routine capture of patient-reported outcome measures (PROMs) and patient-reported experience measures (PREMs) is championed as core infrastructure for learning health systems and value-based care. Yet, the guidance on how to implement these measures is scattered. We synthesised evidence on implementation strategies used to introduce and sustain PROMs and PREMs, and examined how these strategies align with common barriers and stages of implementation.

**Methods:**

We conducted a secondary analysis of an umbrella review (25 reviews; 1086 primary studies, 2014–2023) that catalogued implementation determinants and processes of PROMs and PREMs. Two reviewers independently coded implementation strategies using the 73-item Expert Recommendations for Implementing Change (ERIC) taxonomy. Strategies were temporally mapped to the phases of the Exploration–Preparation–Implementation–Sustainment (EPIS) framework, and onto the barriers identified in the parent review using the CFIR × ERIC matching tool.

**Results:**

Twenty of 25 reviews reported at least one implementation strategy, yielding 152 instances coded to 43 of 73 ERIC strategies. Pre-implementation strategies (74 instances) focused on local consensus building, readiness and barrier assessments, early IT integration, and front-loaded education and champion preparation. During implementation and sustainment (78 instances), the strategies most often used were audit and feedback, real-time data feedback to clinicians, reminders, facilitation, technical assistance, refresher training, and patient onboarding and prompts. Mapping strategies to key barriers showed reasonable coverage for workflow, staff capability, and organisational fit, but gaps for patient capability, long-term financing, data analytics, and equity. Thirty ERIC strategies were not identified, most relating to policy, financing, or market-shaping.

**Conclusion:**

Implementing PROMs and PREMs in routine care requires coordinated changes in relationships, workflows, technology, and incentives. This study organises existing evidence into practical tools that health system teams and researchers can use to select, sequence, and resource implementation strategies for PROM and PREM programmes.

## Introduction

Routine use of patient-reported outcome measures (PROMs) and patient-reported experience measures (PREMs) is a cornerstone of learning health systems and value-based health care because the data offer a real-time window on what matters to patients and where care is falling short [1, 2]. Administered in paper format or electronically, PROMs are validated instruments that aim to capture patients’ own assessments of symptoms, functional status, and quality of life [1, 2], while PREMs aim to assess the interpersonal and organisational aspects of care [3]. When PROM data are fed back to clinicians in a timely, actionable format, they have been linked to earlier symptom detection, fewer emergency visits and better disease control, while PREM data can help teams target communication, coordination and equity gaps [4, 5]. When PROM and PREM results are disaggregated by age, ethnicity, and socioeconomic status, organisations can track care disparities from the patient perspective, strengthening institutional accountability [6, 7].

Early adoption of PROMs occurred mainly in hospitals and specialised oncology programmes [8–10]. Global efforts now aim to embed routine PROMs and PREMs in a wide range of settings and contexts, including gender-affirming care [11], maternity care [12], mental health care [13], primary care [14, 15], and substance use care [16]. The problem is no longer whether PROMs and PREMs add value, but how to embed them in health systems efficiently, equitably and sustainably [10]. This requires addressing multilevel implementation barriers. Patients may lack the health literacy, language support or digital access needed to complete electronic surveys, while clinicians cite appointment time constraints, fragmented data flows and uncertainty about interpreting and acting on scores [10, 14, 17, 18]. Organisations often struggle with non-standardised measure sets, limited electronic health record (EHR) interoperability and competing strategic priorities [10, 14, 17, 18].

Implementation strategies are methods used to adopt or sustain healthcare practices; they address barriers and leverage facilitators to implementation [19–21]. However, guidance on *which* implementation strategies to select and in *what* sequence remains fragmented. Most syntheses to date catalogue barriers (e.g., literacy, data-integration, clinician scepticism) rather than the concrete actions to overcome them [11–15]. Implementers often still rely on ad-hoc methods, such as checklists or local trial-and-error, approaches that waste resources and widen variation in quality and equity. Consolidating the scattered evidence on implementation strategies and mapping those strategies to the typical lifecycle of a PROM and PREM programme could shorten the design phase of these programmes, reduce duplication and accelerate spread.

The Expert Recommendations for Implementing Change (ERIC) taxonomy was developed via a multi-round Delphi process involving over 70 implementation experts and has become a widely adopted standard to improve consistency in the reporting and synthesis of implementation strategies. It provides a common language for 73 discrete strategies grouped into nine functional clusters [22]. The Exploration–Preparation–Implementation–Sustainment (EPIS) framework is a widely cited process model that provides a temporal lens on implementation (Exploration, Preparation, Implementation, Sustainment), highlighting that optimal strategies shift as programmes move from planning to routine use [23]. Combining the ERIC taxonomy and EPIS framework when investigating implementation strategies could provide guidance not only on which implementation strategies to use but also when and in what sequence. This could produce phase-specific, evidence-informed guidance for PROM and PREM implementation and scale-up.

This team has completed a review that explored the barriers and enablers to the implementation of PROMs and PREMs using the Consolidated Framework for Implementation Research (CFIR), one of the most widely used determinant frameworks [24–26]. This secondary analysis builds on that previous work to: (1) identify the implementation strategies reported across reviews and classify them by ERIC taxonomy and implementation phase, (2) map those strategies to the strongest evidence-based barriers to show which strategies address which implementation problems (and where gaps remain), and (3) produce an EPIS-informed roadmap that health care teams can use to plan, resource, and sequence PROMs and PREMs programmes.

## Methods

We conducted a secondary analysis of our previously completed umbrella review [25], which examined multilevel determinants of PROM and PREM implementation, following Joanna Briggs Institute (JBI) guidance for umbrella reviews [27, 28]. While the parent review sought to catalogue barriers and enablers, this follow-up study focuses on how implementers take action in practice. Using a convergent mixed-methods design, we re-reviewed all included articles to identify, classify, and synthesise reported implementation strategies. Quantitative mapping and frequency counts were integrated with qualitative summaries to capture both the breadth and nuance of strategy use.

### Data source and eligibility

This secondary analysis drew on the dataset from an umbrella review (PROSPERO CRD42023421845) with methodological details reported in the published protocol [25]. The parent review included 25 evidence syntheses (systematic, scoping, and narrative reviews) published between 2014 and 2023. Eligible reviews examined implementation processes, barriers, or facilitators related to PROM and PREM use in adult healthcare settings. Reviews focused exclusively on psychometric properties of PROMs and PREMs were excluded. Literature was identified through comprehensive database searches (June 2023), supplemented by forward and backward citation tracking. All screening, data extraction, and quality appraisal were conducted in duplicate by independent reviewers, with conflicts resolved through discussion. Review quality was assessed using the JBI umbrella review checklist.

### Data extraction procedure

For this secondary analysis, we systematically re-examined all included reviews to extract any sentence(s) or clause(s) describing a deliberate action intended to initiate, routinise, or sustain PROM or PREM use. Each identified action was coded using the ERIC taxonomy, a consensus-based framework comprising 73 discrete implementation strategies, each defined and grouped into nine functional clusters [22]. A coding framework was developed in NVivo 14 (QSR International, Burlington, MA, USA) to operationalise the ERIC taxonomy. Strategies were temporally mapped to the pre-implementation phase (actions occurring before go-live, such as planning, training, or IT build) or to the implementation/sustainment phase (actions coinciding with or following the start of routine data capture and clinical use).

All full-text articles and supplementary materials were imported into NVivo. Two researchers independently coded each excerpt to one of the 73 ERIC strategies and its corresponding cluster, maintaining a detailed audit trail. When a passage described multiple distinct actions (e.g., “audit and feedback dashboards and academic detailing”), it was split into separate records to prevent double counting. Coding discrepancies were resolved through discussion or, if needed, adjudicated by a third reviewer. The final reconciled dataset was exported into Microsoft Excel for data cleaning, deduplication, and quantitative synthesis by ERIC domain and strategy.

### Data analysis

All coded sentences or clauses were first exported from NVivo into a single Excel workbook. Each row contained the verbatim excerpt, review identifier, ERIC strategy label and the temporal tag. Excerpts containing multiple actions were split into separate rows, and strategy labels were normalised using data-validation lists. We generated cross-tabulations of strategies by review and by EPIS phase and produced heat maps in R to visualise coverage across ERIC clusters. We mapped all unique strategies to the 15 CFIR barriers with the highest evidence score, i.e., a score based on the frequency of the barriers in the included sources moderated by the quality of the evidence, from the parent umbrella review using the CFIR–ERIC matching tool [29]. This tool was developed in 2019 by surveying implementation experts and maps CFIR barriers to specific implementation strategies in the ERIC taxonomy.

During extraction, strategies were initially coded into two EPIS-informed phases, pre-implementation (Exploration/Preparation combined) and implementation/sustainment, reflecting how activities were reported. For the roadmap (Figure 2), we later separated Implementation and Sustainment to better mirror EPIS and highlight sustainment planning. Quantitative summaries in Tables 3 and 4 therefore retain the combined implementation/sustainment category for analytic robustness, whereas the roadmap disaggregates these phases for end-user guidance. We sequenced strategies within phases based on frequency across reviews, strength of barrier alignment, and dependency logic (e.g., IT build → data relay → audit-and-feedback). The roadmap was iteratively refined by the author team to maximise clarity, usability, and fidelity to the underlying evidence.

## Results

The parent umbrella review included 25 reviews (18 systematic, 6 scoping, 1 umbrella). The characteristics of included reviews are reported in Table 1. These reviews synthesized 1086 unique primary studies. The included reviews spanned diverse clinical contexts, most commonly oncology (*n* = 15), and covered PROMs (*n* = 21), PREMs (*n* = 1), or both (*n* = 3). Most focused on the perspectives of adult, condition-specific populations; 11 also examined provider perspectives. Implementation settings ranged from acute care to community and home-based environments. Methodological quality, assessed with the 19-item JBI checklist, ranged from 9 to 19 (median: 14); 80% of reviews scored ≥13, indicating moderate-to-high quality, while four reviews scored < 12 and were therefore appraised as having substantial methodological limitations.

**Table 1:** Characteristics of included reviews (*N* = 25)

Study (Year)	Review design	**N of included studies and designs**^**†**^	PROMs or PREMs	**Settings**^**‡**^	**Quality**^**§**^
Antunes et al (2014) [30]	Systematic review	31; Qual 12; Obs 10; Other 9	PROMs	Palliative/hospice; Oncology clinics; Hospitals; Multi-setting	14/19
Boyce et al (2014) [31]	Systematic review	16; Qual 16	PROMs	Primary care; Hospital; Mental health; Palliative; Multi-setting	14/19
Breckenridge et al (2015) [32]	Systematic review	9; Obs 4; Rev 2; Other 3	PROMs	Chronic disease registries	9/19
Briggs et al (2020) [33]	Systematic review	15; Obs 12; Exp 2; Other 1	PROMs	Primary care rehab; Outpatient therapy; Multi-setting	16/19
Campbell et al (2021) [34]	Systematic review	52; Qual 50; Mix 2	PROMs	Primary care/outpatient; Hospital; Palliative care; Multi-setting	14/19
Carfora et al (2022) [35]	Systematic review	14; Qual 11; Mix 3	PROMs	Outpatient clinics; Primary care; Physiotherapy; Other outpatient	16/19
Chen et al (2023) [12]	Systematic review	24; Qual 15; Quant 8; Other 1	PROMs	Outpatient maternity clinics	15/19
Dorr et al (2022) [36]	Systematic review	16; Exp 10; Obs 3; Other 3	PROMs	Surgical departments; Primary care; Other hospital settings	14/19
Foster et al (2018) [37]	Umbrella review	6; SR 3; Realist 1; Other 2	PROMs	Palliative care; Cancer services; Other environments	15/19
Gelkopf et al (2021) [13]	Systematic review	103; Not reported	PROMs	Outpatient mental health; Inpatient units; Other settings	10/19
Glenwright et al (2023) [38]	Systematic review	24; Qual 13; Mix 7; Other 4	ePROMs/ePREMs	Oncology; Community health; Hospital wards; Other settings	18/19
Howell et al (2015) [39]	Scoping review	34; Exp 8; Feas 5; Other 21	PROMs	Cancer clinical practice settings	12/19
Kamran et al (2023) [11]	Systematic review	286; OutRes 190; Obs 94; Other 2	PROMs	Outpatient gender-affirming care clinics	15/19
Kynoch et al (2022) [40]	Scoping review	86; Quant 56; Qual 11; Other 19	PROMs/PREMs	Inpatient wards; Outpatient clinics; EDs; Oncology; Other settings	13/19
Lopez et al (2023) [41]	Scoping review	63; Exp/QI 38; Obs 6; Other 2	ePROMs	Active treatment & follow-up clinics; Post-op units; Palliative care	13/19
Lutz et al (2022) [42]	Systematic review	25*; Feas 11; Prot 6; Other 8	PROMs	Oncology practice settings	13/19
Meirte et al (2020) [43]	Systematic review	32; Obs 14; Exp 18	ePROMs	Outpatient clinics; Home-based assessments; Other settings	15/19
Migchels et al (2023) [16]	Scoping review	23; Obs 16; Mix 4; Other 3	PROMs/PREMs	Outpatient centres; Residential programmes; EDs; Other settings	13/19
Minvielle et al (2023) [44]	Scoping review	68**; Obs 15; Rev 12; Other 41	PROMs	Oncology; Palliative care units; Surgical units; Other settings	15/19
Nguyen et al (2021) [17]	Systematic review	14; Qual 7; Obs 4; Other 3	PROMs/PROs	Cancer centres; Palliative care units; Other settings	10/19
Nic Giolla Easpaig et al (2020) [45]	Systematic review	34; Mix 17; Qual 17	PROMs	Cancer clinics; Hospitals; Primary care; Community; Other settings	15/19
Nielsen et al (2020) [46]	Scoping review	51; Obs 21; Exp 12; Other 18	ePROMs	Outpatient care settings	13/19
Shunmuga Sundaram et al (2022) [18]	Systematic review	20; Qual 11; Mix 9	PREMs	Hospital units; Primary care settings; Other environments	13/19
van Egdom et al (2019) [47]	Systematic review	34; Obs 15; Exp 14; Other 5	PROMs	Oncology clinics	15/19
Yang et al (2018) [48]	Systematic review	43; Exp 16; Qual 15; Other 12	PROMs	Oncology clinics; Hospital/general clinics; Other settings	19/19

^†^Only top two designs listed, numbers indicate how many included studies featured each group; Qual = qualitative; Obs = observational; Exp = experimental; QI = quality improvement; Feas = feasibility; Rev = review; Mix = mixed methods; OutRes = outcomes research; SR = systematic review; Prot = protocol; NR = not reported

^‡^Only top four categories listed

^§^Quality score out of 19 items (JBI Critical Appraisal Checklist)

*Lutz et al. extracted data from 25 of 63 identified articles after quality screening

**Minvielle et al. also included 19 reports

### Distribution of strategies to support the implementation of PROMs and PREMs

Twenty of the 25 reviews described at least one action designed to promote, execute, or sustain PROM or PREM use. We were unable to identify such actions in five reviews [16, 31, 34, 35, 46]. Across the 20 reviews, we coded 152 discrete actions to 43 of the 73 ERIC implementation strategies (58.9%). Reviews reported between 1 and 18 strategies each (median: 7; interquartile range: 5–11).

All nine ERIC clusters were represented (Table 2). Complete or near-complete coverage was observed in the clusters *Provide interactive assistance* (4/4 strategies), *Adapt and tailor to context* (3/3), and *Use evaluative and iterative strategies* (9/10), underscoring the prominence of strategies focusing on feedback, adaptation, and hands-on facilitation. Coverage was moderate for *Develop stakeholder inter-relationships* (11/17), *Train and educate stakeholders* (5/11), *Support clinicians* (3/5), and *Engage consumers* (3/5). The least populated clusters were *Use financial strategies* (3/9) and *Change infrastructure* (2/8). Definitions of ERIC clusters are provided in Table 2.

**Table 2:** ERIC clusters represented in the updated evidence map across the 20 reviews reporting implementation strategies

ERIC cluster	Concise cluster definition *	Strategies identified/total in cluster	Reviews with ≥ 1 strategy †
**1. Use evaluative and iterative strategies**	Use data to diagnose problems, track progress, and refine implementation	9/10	11
**2. Provide interactive assistance**	Offer ongoing, hands-on support (facilitation, tech help, supervision)	4/4	9
**3. Adapt and tailor to context**	Modify innovation or strategies to fit local barriers/facilitators	3/3	7
**4. Develop stakeholder inter-relationships**	Build or leverage relationships among people or organisations	11/17	12
**5. Train and educate stakeholders**	Improve knowledge and skills via meetings, materials, ongoing training	5/11	15
**6. Support clinicians**	Provide tools, data, reminders, or role changes that ease uptake	3/5	14
**7. Engage consumers**	Engage or partner with patients/families to enhance uptake	3/5	10
**8. Use financial strategies**	Employ funding, fees, or contracts to influence implementation	3/9	5
**9. Change infrastructure**	Alter structures, equipment, or records to accommodate innovation	2/8	7

*Definitions paraphrased from Powell et al. [22] ERIC taxonomy

^†^Number of reviews (out of 20) that reported at least one strategy in the cluster

**Fig. 1:**
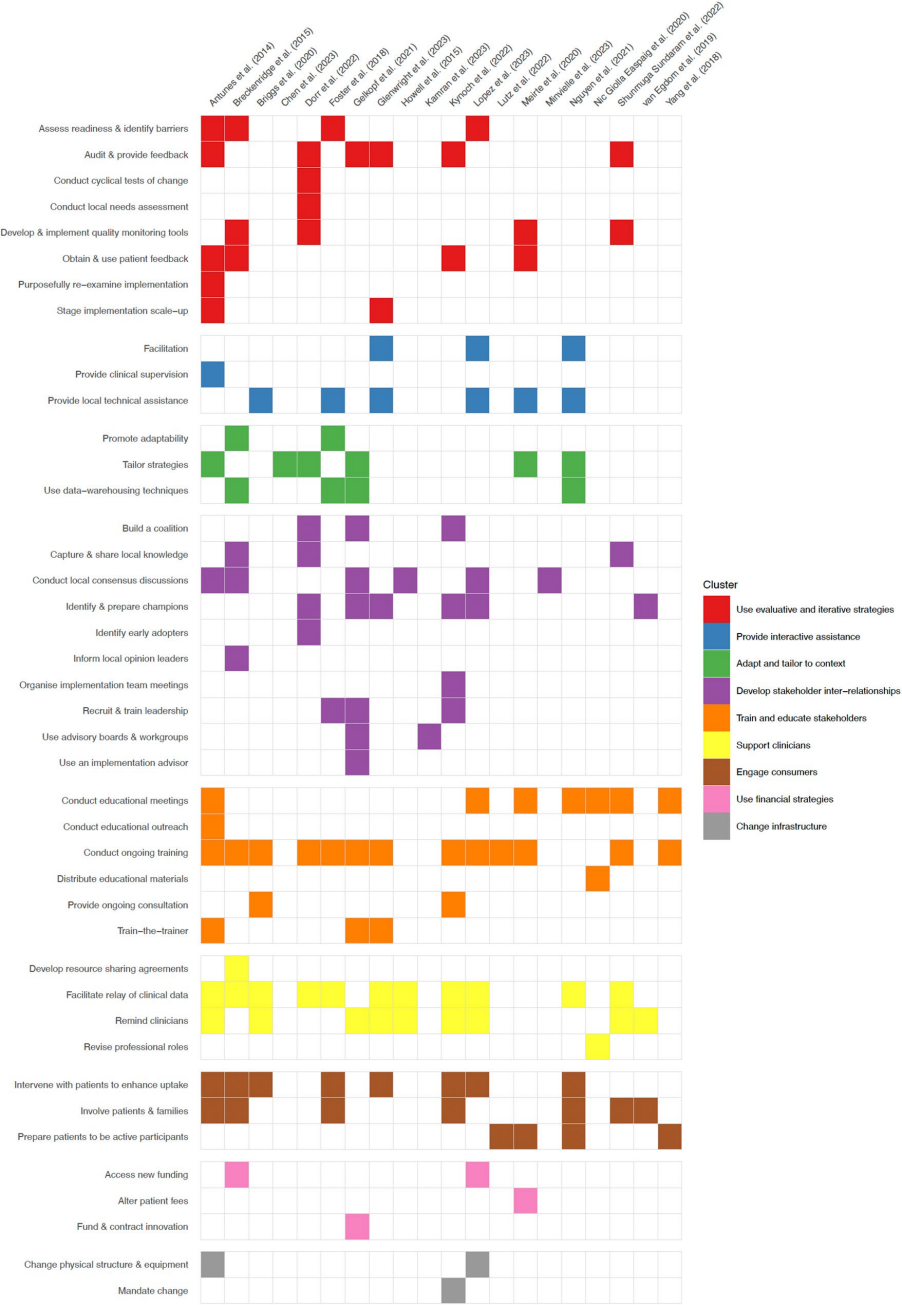
ERIC implementation strategies reported across included reviews, organised by cluster and review

Figure 1 presents the distribution ERIC implementation strategies reported across the 20 reviews, organised by cluster and review. At the individual-strategy level, 14 implementation strategies accounted for more than half of all coded actions. *Conduct educational meetings* was cited most frequently (13 reviews), followed by *Facilitate relay of clinical data to providers* (10), *Remind clinicians* (9), *Audit and provide feedback* (7), *Conduct local consensus discussions* (7), *Intervene with patients/consumers to enhance uptake and adherence* (7), *Involve patients/consumers and family members* (6), *Obtain and use patient/consumer and family feedback* (6), *Conduct ongoing training* (6), *Assess for readiness and identify barriers and facilitators* (5), *Develop and organize quality monitoring systems* (5), *Facilitation* (5), *Tailor strategies* (5) and *Change record systems* (5). No other implementation strategy appeared in five or more reviews.

### Strategies used in the pre-implementation, implementation and sustainment phases of PROM and PREM initiatives

A total of 74 strategy excerpts were coded to the pre-implementation phase, encompassing exploration and preparation for implementation, across the 20 reviews. Eight of the nine ERIC clusters of strategies were represented. Table 3 summarises the 17 implementation strategies that were cited in at least two reviews for the pre-implementation phase.

**Table 3:** Implementation strategies most frequently cited (≥2 reviews) for the pre-implementation phase of PROM and PREM initiatives

Implementation strategy	ERIC cluster	Concise definition*	PROM/PREM-specific application (pre-implementation)	N	Reviews
**Conduct educational meetings**	Train and educate stakeholders	Hold meetings for different stakeholder groups to teach them about the clinical innovation	• Front-loaded sessions to build clinicians’ knowledge and skills in selecting, administering, interpreting, and communicating PROM and PREM data • Content should span PROM and PREM psychometric properties, communication skills, role-play, and integration of results into care, tailored to local context	13	[13, 17, 18, 30, 32, 36, 37, 40–43, 45, 48]
**Conduct local consensus discussions**	Develop stakeholder inter-relationships	Engage local providers and stakeholders to decide whether the problem and proposed innovation are important and appropriate	• Early consensus meetings with clinicians, staff, and leadership to explore feasibility, co-design PROM and PREM implementation plans and workflows, and foster shared ownership • Transparent goal setting, data collection, and evaluation processes build trust, reduce resistance, and foster shared ownership	7	[13, 30, 32, 36, 37, 40, 41]
**Assess for readiness and identify barriers and facilitators**	Use evaluative and iterative strategies	Evaluate organisational readiness, potential barriers, and available strengths	• Appraise resources, staff capacity, and contextual factors; map barriers and facilitators to implementation and sustainability • Judge feasibility of candidate PROMs and PREMs and devote time to align instruments and workflows with local conditions	5	[30, 32, 37, 40, 41]
**Change record systems**	Change infrastructure	Modify information systems to improve implementation or clinical care	• Integrate ePROM and ePREM workflows into EHRs: automated data capture, alerts, and pathway triggers driven by PROM scores • Requires investments in databases, IT infrastructure, admin support, and adaptation of legacy systems (e.g., cancer EMRs)	5	[13, 17, 37, 38, 43]
**Identify and prepare champions**	Develop stakeholder inter-relationships	Enlist and prepare key individuals to drive and sustain implementation	• Select influential clinicians and staff to advocate for PROM and PREM value, model use, and motivate peers • Champions support patient acceptance and facilitate peer-to-peer learning throughout rollout	4	[36, 38, 41, 47]
**Prepare patients or consumers to be active participants**	Engage consumers	Equip patients to engage actively in their care and inquire about evidence-based decisions	• Train patients on device/platform use, clarify purpose and benefits of PROM and PREM collection, and coach interpretation of results • Public-facing campaigns generate interest and encourage meaningful, informed participation	4	[17, 32, 42, 48]
**Obtain and use patients/consumers and family feedback**	Use evaluative and iterative strategies	Develop methods to elicit and act on patient and family feedback about implementation	• Involve patients in defining objectives, data collection processes, and reporting formats • Patient perspectives should inform PROM and PREM selection and ensure relevance, acceptability, and usability	3	[32, 39, 40]
**Obtain formal commitments**	Develop stakeholder inter-relationships	Secure written or formal agreements from key partners specifying their implementation roles	• Finalise legal data-sharing agreements • Obtain senior-level commitments for ongoing organisational support (e.g., protected staff time, resources) and budgets for IT upgrades, databases, and dedicated admin resources	3	[30, 32, 37]
**Use advisory boards and workgroups**	Develop stakeholder inter-relationships	Create and/or engage a multi-stakeholder group to provide ongoing input and advice	• Form committees with clinicians, administrators, and patient organisations to guide PROM and PREM rollout • Leverage patient groups to advocate with policymakers and refine strategy	3	[11, 32, 40]
**Distribute educational materials**	Train and educate stakeholders	Provide guidelines, manuals, or toolkits in person, by mail or electronically	• Host web-based resources (toolkit plus quick-reference handouts) on PROM and PREM use • Embed concise guidelines and job-aids within training sessions and EHR portals	3	[17, 33, 45]
**Recruit, designate, and train for leadership**	Develop stakeholder inter-relationships	Recruit, designate, and train leaders for the change effort	• Designate clinical/managerial leads trained in cascade communication and data-driven decision-making • Leaders coordinate feedback loops and adapt implementation strategies	3	[13, 30, 40]
**Develop a formal implementation blueprint**	Use evaluative and iterative strategies	Produce a written plan detailing goals, timelines, roles, and strategies	• Draft an implementation roadmap covering governance, data flow, training, and evaluation milestones • Include a preparatory checklist and benchmarking plan for participating sites	2	[36, 37]
**Promote adaptability**	Adapt and tailor to context	Clarify which innovation elements may be tailored and which must remain fixed	• Permit site-level flexibility (e.g., ePROM modality) while preserving core measurement fidelity • Embed iterative adaptation processes to fit diverse patient and organisational needs	2	[32, 37]
**Develop academic partnerships**	Develop stakeholder inter-relationships	Partner with a university or academic unit for shared training and research support	• Collaborate with academics to select validated PROM and PREM instruments and optimise protocols • Establish ongoing analytic support through research/audit group linkages	2	[13, 30]
**Use an implementation advisor**	Develop stakeholder inter-relationships	Seek guidance from experts in implementation	• Engage external implementation experts to advise on design, rollout, and fidelity monitoring • Contract consultancies to troubleshoot barriers and scale best practices	2	[13, 32]
**Access new funding**	Use financial strategies	Access new or existing money to facilitate the implementation	• Obtain grants or organisational funds for licensing, IT upgrades, and training costs • Use financial incentives to accelerate adoption and sustain PROM and PREM use	2	[32, 41]
**Change physical structure and equipment**	Change infrastructure	Reconfigure space and/or procure equipment to accommodate the innovation	• Install kiosks/tablets and upgrade workstations to enable on-site ePROM and ePREM completion • Enhance network and hardware capacity for real-time data capture and analysis	2	[30, 41]

#### Pre-implementation phase

The most frequent pre-implementation strategies focused on building and formalising relationships among key stakeholders (26/74 excerpts; 35%). Seven reviews described local consensus meetings in which clinicians, managers and often patient representatives met early to explore feasibility and readiness, agree on clear goals and co-design data-collection workflows; these meetings were credited with reducing resistance and cementing shared ownership of PROM and PREM roll-out [8, 32, 36–38, 46]. Four reviews highlighted the importance of identifying and preparing champions, typically respected nurses, physicians or therapists who were briefed to model use of PROMs and PREMs, answer colleagues’ questions and reassure patients [36, 38, 41, 47]. Three reviews discussed securing formal commitments such as signed data-sharing agreements and written pledges of protected time, IT budgets and administrative support, signalling top-level endorsement [30, 32, 37]. Advisory boards or workgroups featuring clinicians, operational leads and patient-organisation members were reported in another three reviews, providing an ongoing forum to refine strategy and, in some cases, lobby policymakers [11, 32, 40]. Similarly, three reviews described recruiting and training clinical or managerial leads responsible for cascading information and coordinating feedback loops [13, 30, 40].

The next-most frequent pre-implementation strategies focused on training and education (16/74 excerpts; 22%). Fourteen reviews described educational meetings before “go-live,” delivered as half-day workshops or brief modular sessions [13, 17, 18, 30, 32, 36, 37, 40–43, 45, 48]. Content typically covered (i) choosing the right PROM or PREM for the context and patient population, (ii) practical tips for electronic, kiosk or paper administration, (iii) interpreting scores, including clinically important change thresholds, and (iv) communicating results with patients to guide shared decision-making. Many programmes paired didactic teaching with hands-on practice (e.g., mock consultations, dashboard walk-throughs) so that clinicians left with the skills to embed the measures in routine visits.

A smaller but important share of pre-implementation work centred on evaluative and iterative strategies, essentially the “measure-learn-adjust” cycle of implementation (11/74 excerpts; 14.9%). Five reviews described assessing readiness and barriers, systematically mapping resources, IT capacity, staffing, and contextual determinants to judge feasibility and identify early fixes [30, 32, 37, 40, 41]. Three reviews reported obtaining and using patient and family feedback before launch, via interviews, focus groups, or pilot surveys, to ensure that chosen measures, wording, and reporting formats were acceptable and useful [32, 39, 40].

Financial, adaptation, and explicit clinician-support strategies were comparatively uncommon in this phase. Financial strategies appeared in 4/74 excerpts (5%) across four reviews, typically referring to securing new or dedicated funding for PROM and PREM initiatives or emphasising that ePROMs should be free for end-users [13, 30, 34, 36]. Adapt and tailor to context was described in 3/74 excerpts (4%), mainly through promoting adaptability of survey mode and timing while protecting core measurement fidelity and embedding “adapt-and-learn” loops into roll-out plans [36, 38, 46]. Finally, only two reviews mentioned strategies related to supporting clinicians (2/74 excerpts; 2%): one emphasised clarifying roles and responsibilities for the management of care [32], and the other described how clinicians incorporated PROMs into their workflows [38].

#### Implementation and sustainment phases

Seventy-eight strategy excerpts were coded to the implementation and sustainment phase, with seven of nine ERIC clusters represented. Table 4 summarises the 15 strategies cited in at least two reviews for this phase.

**Table 4:** Implementation strategies most frequently cited (≥2 reviews) for the implementation and sustainment phase of PROM and PREM initiatives

Implementation strategy	ERIC cluster	Concise definition*	PROM/PREM-specific application (Implementation and sustainment)	N	Reviews
**Facilitate relay of clinical data to providers**	Support clinicians	Design and use mechanisms that give clinicians timely, actionable patient-level data to inform care	• Provide real-time, longitudinal PROM and PREM data stratified by relevant subgroups with clear thresholds, automated alerts for concerning scores, and user-friendly displays accessible to all authorised staff • Make anonymised datasets freely shareable across clinical teams to support care coordination, benchmarking, and quality improvement, within established confidentiality constraints.	9	[17, 18, 32, 36, 38–41, 43]
**Remind clinicians**	Support clinicians	Deliver prompts that cue clinicians to perform the desired action	• Deploy email, EHR pop-ups, and online follow-up reminders that flag clinically important PROM and PREM scores, cue clinicians to discuss results during consultations, and nudge patients or staff to complete ePROM and ePREM surveys • Use standing goals and peer-to-peer prompts on wards to maintain high distribution and completion rates, embedding reminder routines into workflows	9	[13, 18, 30, 33, 38–41, 47]
**Audit and provide feedback**	Use evaluative and iterative strategies	Collect data over a specified period, summarise it, and feed it back to clinicians or managers to encourage behaviour change	• Audit and use dashboards to share patient-level progress data, responder vs non-responder profiles, and cross-hospital benchmarks • Pair audit results with coaching to adjust workflows, spotlight positive PROM and PREM scores to reinforce good practice, and strengthen clinicians’ commitment	7	[13, 18, 30, 32, 36, 38, 40]
**Intervene with patients or consumers to enhance uptake and adherence**	Engage consumers	Strategies directed at patients or caregivers to increase initiation of and adherence to recommended care	• Provide printed or electronic leaflets, quick tutorials, and one-to-one support that show patients how to use ePROMs, emphasise data security, and stress that routine care will not be disrupted • Highlight the direct benefits of participation and send scheduled prompts (e.g., email/SMS) to encourage timely completion and sustained engagement	7	[17, 30, 32, 33, 37, 38, 41]
**Conduct ongoing training**	Train and educate stakeholders	Provide continuing education or skill-building sessions after initial rollout	• Run periodic refresher seminars or guided workshops • Should update staff on collecting, analysing, and interpreting PROM and PREM data, reinforcing skills and confidence	6	[13, 32, 33, 36, 38, 40]
**Facilitation**	Provide interactive assistance	Supply interactive problem-solving and support to help individuals or teams overcome barriers	• Assign coordinators or super-users who maintain strong relationships across teams, troubleshoot workflows, and supply the resources needed for data capture/analysis • On-site or virtual coaching that helps clinicians interpret PROM and PREM results and refine processes	5	[17, 30, 37, 38, 41]
**Involve patients/consumers and family members**	Engage consumers	Engage patients and families as partners in planning, decision-making, or evaluation	• Give patients the option to complete PROMs and PREMs on their own devices at convenient times and share results with caregivers • Review PROM findings jointly with patients and invite them to pinpoint priority areas for change	5	[17, 18, 30, 40, 43]
**Develop and organize quality monitoring systems**	Use evaluative and iterative strategies	Develop and organize systems that monitor processes and outcomes for quality assurance and improvement	• Collect and refresh survey data at regular intervals, generate longitudinal trend reports, and benchmark hospitals or clinicians against peers • Feed performance metrics and comparative dashboards into quality-improvement cycles, guiding targeted changes and tracking impact over time	4	[18, 36, 38, 40]
**Provide local technical assistance**	Provide interactive assistance	Offer hands-on, context-specific help to troubleshoot problems and build local capacity	• Give users direct email/phone access to project managers or support staff for practical and technical issues with the ePROM or ePREM app • Supply immediate guidance after use	4	[33, 38, 41, 43]
**Tailor strategies**	Adapt and tailor to context	Deliberately adjust implementation strategies to address identified barriers or align with local culture, resources, and workflows	• Adapt workflows and even the survey format (e.g., paper vs electronic) to each organisation’s resources and response patterns. • Choose PROMs and PREMs that capture the data you need and tailor question sets or explanatory information for individual patients to boost relevance/engagement	4	[12, 17, 44, 47]
**Obtain and use patients /consumers and family feedback**	Use evaluative and iterative strategies	Strategies to increase patient and family feedback on the implementation effort	• Review ePROM results with patients (or proxies when direct communication isn’t possible) • Assign a coordinator to log patient or family complaints and resolve issues immediately, feeding lessons back into process improvements.	3	[17, 30, 43]
**Conduct cyclical small tests of change**	Use evaluative and iterative strategies	Implement changes in a cyclical fashion using small tests of change before taking changes system-wide	• Conduct small cyclical tests of implementation of PROMs and PREMs to refine processes • Refine processes to obtain better quality data	2	[13, 36]
**Develop and implement tools for quality monitoring**	Use evaluative and iterative strategies	Develop, test, and introduce protocols, algorithms, standards, and measures for quality monitoring	• Plot results with a graph or visual aids and give both patients and clinicians better insight into patients’ health status • Tools to link clinical outcome measures to PROMs	2	[36, 43]
**Stage implementation scale-up**	Use evaluative and iterative strategies	Phase implementation efforts by starting with small pilots before a larger rollout	• Start with small-scale projects • Implementation in stages to improve acceptability	2	[30, 36]
**Use train-the-trainer strategies**	Train and educate stakeholders	Prepare a core group of experts who then teach and mentor additional staff	• Provide train-the-trainer models • Enable peer-to-peer learning	2	[13, 38]

During active roll-out and into sustainment of PROMs and PREMs, organisations leaned most heavily on evaluative and iterative strategies (21/78 excerpts; 27%). The core strategy was regular audit and feedback: seven reviews described extracting PROM and PREM data at scheduled intervals, displaying patient outcomes and trajectories and peer benchmarks on dashboards, and pairing the numbers with brief coaching huddles so teams could tweak workflows and celebrate gains [13, 18, 30, 32, 36, 38, 40]. Four reviews suggested going further by building continuous-monitoring systems automating pulls that refreshed run charts and heat maps directly into quality improvement meetings, effectively treating PROM and PREM scores like any other key performance indicator [18, 36, 38, 40].

The next-most common strategies in the roll-out and sustainment phase focused on directly supporting front-line clinicians (18/78 excerpts; 23%). Nine reviews described building pipelines that relay PROM and PREM results back to providers in real time: longitudinal dashboards stratified by disease stage or risk group, colour-banded thresholds and automated alerts for worrisome scores were embedded in the EHR or a linked portal, while anonymized, service-level datasets were made available for care-team huddles, benchmarking and quality-improvement projects [17, 18, 32, 36, 38–41, 43]. Another nine reviews described implementing reminder systems including emails, EHR pop-ups and ward-level notice boards that cued staff to check new scores, discuss findings during the visit, or prompt patients to complete overdue surveys [13, 18, 30, 33, 38–41, 47].

Patient-focused strategies were also prominent during roll-out (12/78 excerpts; 15%). Seven reviews reported efforts to intervene with patients to boost uptake and adherence: teams supplied quick-start leaflets or video tutorials, offered one-to-one help at kiosk or bedside, assured patients that data were secure and would not lengthen appointments, and sent scheduled e-mails or SMS reminders to sustain survey completion [17, 30, 32, 33, 37, 38, 41]. Five reviews suggested going further to involve patients and families as active partners, allowing them to fill in PROMs and PREMs on their own devices, reviewing results in real time with clinicians or caregivers, and helping prioritise which issues should trigger follow-up [17, 18, 30, 40, 43].

A further 11 of the 78 in-use excerpts (14%) described strategies to provide interactive assistance to clinicians. Five reviews spoke of formal facilitation: services appointed a “super-user” or implementation coordinator who built strong cross-team relationships, offered on-site or virtual coaching, and helped clinicians interpret scores, adjust alert thresholds and streamline data entry [17, 30, 37, 38, 41]. Four reviews added local technical assistance in the form of a dedicated phone line or e-mail address staffed by project managers or IT analysts who could resolve log-in glitches, broken survey links or dashboard display errors within hours rather than weeks [33, 38, 41, 43].

Training and educating stakeholders, adapting and tailoring to context, and stakeholder interrelationships were the least commonly used strategies during this phase. Nine reviews (9/78 excerpts; 11%) described training and education for stakeholders. Six reviews described periodic refresher seminars or short, guided workshops that updated staff on new dashboards and provided interpretation tips and communication techniques, thereby shoring up confidence and preventing skill-drift [13, 32, 33, 36, 38, 40], two reviews described a train-the-trainer model, preparing a small cadre of super-users who could onboard new hires and provide peer coaching at minimal additional cost [13, 38]. Five reviews (5/78 excerpts; 6%) adapted and tailored to context.

Four reviews reported tailoring implementation after go-live: sites tweaked survey formats (paper vs tablet), streamlined question sets to match local workflows, and added language or condition-specific explanations so that PROM and PREM collection remained relevant and feasible as contexts evolved [12, 17, 44, 47]. Finally, stakeholder interrelationships were the least commonly used strategy with 2 of 78 in-use excerpts (2%). Both reviews made efforts to sustain stakeholder interrelationships such as producing outputs in multiple formats, as well as using regular meetings to collaboratively track and monitor implementation progress [34, 36].

### Mapping implementation strategies to the 15 highest-evidence barriers

Mapping the 43 unique implementation strategies identified across the 20 reviews onto the 15 highest evidence CFIR barriers (Table 5) shows mixed coverage. Common workflow barriers are addressed reasonably well: many programmes upgraded EHRs, built dashboards and changed record systems, but they rarely went further to redesign roles and workflows or automate reminder systems. Capability-building efforts largely target clinicians (education, refresher training, facilitation), with comparatively few strategies aimed at patient capability, such as literacy, language or digital access. Design and complexity issues prompted some local tailoring, yet rigorous co-design, simulation testing and iterative PDSA cycles appear in only a small number of reviews. Motivational levers are also underused: despite evidence that perceived clinical benefit drives uptake, strategies such as opinion leader outreach, patient storytelling or incentives are rarely deployed. Infrastructure investments focus on core EHR functions, leaving gaps in data warehousing, analytics capacity and central technical support, while policy and financing strategies are almost entirely absent, despite sustained funding being a recurrent barrier.

**Table 5:** Systematic CFIR/ERIC mapping illustrating how well the strategies identified cover the 15 highest-evidence barriers to the implementation of PROMs and PREMs identified in umbrella review (numbers in parentheses = N of reviews citing that strategy)

Barrier	CFIR domain and construct	Key problems summarized from reviews	ERIC implementation strategies identified that target the barrier*
**Poor integration of PROMs and PREMs into clinician workflow and high burden**	Inner Setting – Work Infrastructure	Forms not embedded into systems, extra clicks, no hand-off, limited staff/time	• Change record systems (5) • Facilitate relay of clinical data (10) • Develop quality monitoring systems (4) • Revise professional roles (1) • Change physical structure and equipment (2) • Stage implementation scale-up (2)
**Limited patient capability to complete PROMs and PREMs**	Individuals – Innovation Recipients, Capability	Low literacy, language, digital divide	• Prepare patients to be active participants (4) • Intervene with patients to enhance adherence (7) • Distribute educational materials (3) • Tailor strategies (4)
**Poorly designed PROM and PREM tools and platforms**	Innovation – Design	Clunky UI, no graphs, no culture or language fit	• Promote adaptability (2) • Tailor strategies (4) • Patient/family feedback loops (3) • Develop & implement quality monitoring tools (2) • Cyclical small tests of change (2)
**Staff knowledge and skill gaps**	Individuals – Innovation Deliverers, Capability	Clinicians unsure how to administer, interpret and act on PROMs and PREMs	• Conduct educational meetings (13) • Conduct ongoing training (6) • Train-the-trainer (2) • Provide ongoing consultation/facilitation (5) • Provide local technical assistance (4)
**Low staff motivation**	Individuals – Innovation Deliverers, Motivation	Resistance, fear of evaluation	• Identify & prepare champions (4) • Conduct local consensus discussions (7) • Audit & provide feedback (7) • Obtain formal commitments (3)
**Excessive complexity of PROM and PREM tools and platforms**	Innovation – Complexity	Too many items, multiple platforms	• Tailor strategies (4) • Promote adaptability (2) • Cyclical small tests (2) • Develop implementation blueprint (2)
**Lack of training and guidelines**	Inner Setting – Access to Knowledge & Information	No standard operating procedures, ad-hoc teaching	• Conduct educational meetings (13) • Ongoing training (6) • Train-the-trainer (2) • Educational materials (3) • Local technical assistance (4)
**Poor compatibility with existing processes**	Inner Setting - Compatibility	PROM and PREM tasks interrupt workflows	• Local consensus discussions (7) • Tailor strategies (4) • Promote adaptability (2) • Assess readiness & barriers (5) • Provide ongoing consultation/facilitation (5)
**Uncertain clinical benefit and validity**	Innovation – Evidence base	Doubts that PROMs change care and about their psychometric properties	• Educational meetings (13) • Audit & feedback (7) • Develop academic partnerships (2) • Educational materials (3)
**Time burden and opportunity cost**	Individuals – Innovation Deliverers, Opportunity	Extra clicks, longer visits	• Change record systems (5) • Facilitate relay of data (10) • Remind clinicians (9) • Revise professional roles (1) • Access new funding (2)
**Inadequate IT infrastructure**	Inner Setting – IT Infrastructure	Fragmented EHR, no interfaces	• Change record systems (5) • Develop quality-monitoring systems (4) • Provide local tech assistance (4)
**Low perceived value and duplicative**	Innovation – Relative Advantage	PROMs and PREMs perceived as adding no benefits over standard of care	• Consensus discussions (7) • Champions (4) • Educational meetings (13) • Audit & feedback (7)
**Lack of equipment and logistics**	Inner Setting – Materials & Equipment	No tablets, printers, space	• Access new funding (2) • Change physical structure & equipment (2) • Develop resource-sharing agreements (0)
**Generic and inflexible measures**	Innovation – Adaptability	PROMs and PREMs not tailored to context and population	• Tailor strategies (4) • Promote adaptability (2) • Patient/family feedback (3) • Re-examine implementation (1)
**Misalignment with mission and priorities**	Inner Setting – Mission Alignment	PROMs and PREMs seen as extra, not strategic	• Consensus discussions (7) • Recruit & train leaders (3) • Champions (4) • Advisory boards/workgroups (3) • Formal commitments (3)

*ERIC matches derived from the Waltz et al. CFIR and ERIC matching tool

### Strategies not identified in the review

A total of 30 ERIC implementation strategies were not identified in any of the included reviews. Collectively, these “unused” strategies are mostly macro-level policy and financing levers (e.g., changing accreditation, liability laws, payment schemes, service sites, fee-for-service lists), or broad dissemination and market-shaping strategies (e.g., use mass media, increase demand, start a dissemination organization), alongside a set of capacity-building or infrastructure strategies (e.g., create learning collaboratives, promote network weaving, involve executive boards, work with educational institutions, develop resource-sharing agreements, use data experts, modeling/simulation). It is unsurprising that they were not selected: in most implementation projects, teams operate at the level of services, programs, or organizations and have limited authority to change payment models, credentialing, or legislation; these options also tend to be rated as relatively low feasibility.

Several of the high-salience barriers we identified in the parent umbrella review [26] are, in principle, best addressed by the 30 “unused” ERIC strategies precisely because those barriers sit upstream in the outer setting (policy/financing) or require cross-organizational infrastructure that local implementation teams rarely control. For example, the recurrent barriers around affordability and funding (Innovation–Cost: *up-front and recurring costs with no dedicated budget/coverage*; Inner Setting–Funding: *lack of financial resources for continued PROM/PREM collection*) [26] would map most directly to macro levers such as changing payment schemes and reimbursement policies, adding PROM and PREM activities to fee-for-service lists, modifying insurer coverage, or linking PROM and PREM reporting to accreditation requirements, strategies that target the root cause (no sustainable external funding) rather than asking clinics to “do more with the same.” Similarly, barriers related to liability concerns and regulatory complexity (Outer Setting–Policies & Laws: *compliance burden, liability fears, privacy/security concerns*) [26] align with ERIC-type strategies like clarifying and adjusting liability rules, standardizing data-sharing agreements, and creating enabling governance frameworks across jurisdictions. 

On the infrastructure side, persistent barriers such as EMR integration and interoperability failures (Inner Setting–IT Infrastructure) and the need for analytic capacity (e.g., keeping databases current, reporting dashboards) [26] are the kinds of problems that capacity-building strategies could address, e.g., develop resource-sharing agreements across organizations, bring in analytics support, create shared technical platforms, or use simulation to plan workflows and staffing before rollout. Finally, barriers linked to variable knowledge/skills and inconsistent training pipelines (Individuals–Deliverers: *low competence/confidence; lack of knowledge and guidance*) [26] could be more structurally tackled through working with educational institutions, embedding PROM and PREM competencies into curricula/continuing education requirements, and establishing learning collaboratives or network weaving to spread practical know-how across sites. In short, these unused strategies line up with the most complex, system-level barriers, but they were unsurprisingly absent in the included reviews because most PROM and PREM implementation efforts operate at the service/organization level with limited authority and often lower feasibility to change payment models, accreditation, or legislation.

### Evidence-informed roadmap for implementing PROMs and PREMs

**Fig. 2:**
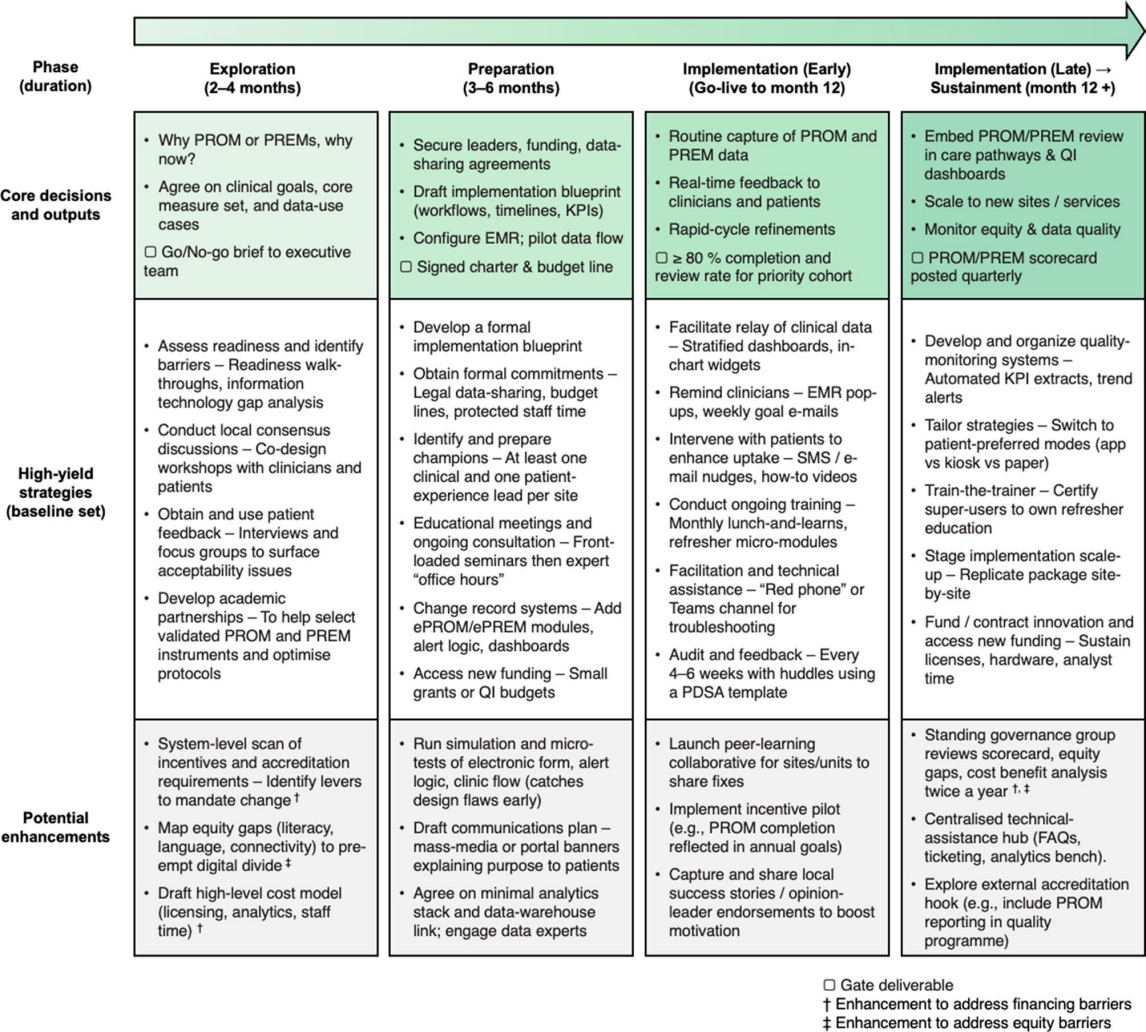
Roadmap for implementing PROM and PREM programmes across the four phases of the EPIS framework

Figure 2 presents an evidence-informed roadmap synthesizing findings from this secondary analysis, presenting the most frequently reported ERIC implementation strategies with the four EPIS phases. In each column, Exploration, Preparation, Early Implementation, and Late Implementation/Sustainment, the roadmap distinguishes three layers: (i) *core decisions and outputs* (items marked ▢ signal “gate” deliverables); (ii) *baseline strategies* that appeared in ≥ 4 reviews for pre-implementation or ≥ 3 reviews for later phases; and (iii) *optional enhancements* drawn from less-common but potentially high-leverage strategies.

## Discussion

We conducted a secondary analysis of an umbrella review dataset to characterise the implementation strategies used to support the uptake of PROMs and PREMs in healthcare. Across 20 of the 25 included reviews that reported implementation strategies, we identified 43 of the 73 ERIC strategies spanning all nine clusters. Pre-implementation strategies were focused on developing stakeholder partnerships, conducting educational workshops, performing readiness assessments, and laying the groundwork through IT system upgrades and basic consumer engagement. As initiatives progressed to the implementation and sustainment phases, strategies increasingly emphasised iterative learning cycles, including audit and feedback, real-time dashboards, clinician-facing alerts and reminders, and technical or facilitative supports. Additional strategies targeted patients through onboarding prompts and adherence reminders, while tailoring and periodic training sessions helped programmes adapt and persist over time.

Taken together, these patterns suggest a phased evolution in implementation strategy use. Pre-implementation efforts tended to be relationship-centred, prioritising trust-building and shared mental models through consensus-building meetings, formal endorsements, and training. This reflects the sociotechnical nature of PROM and PREM integration: while the tools themselves are relatively simple, their routine use demands coordinated shifts in clinical roles, workflows, and accountabilities. By contrast, the implementation and sustainment phases were dominated by data-driven strategies aimed at embedding PROM and PREM use into clinical practice through feedback, nudges, and problem-solving infrastructure. The reliance on just-in-time supports like facilitation, technical assistance, and refresher education underscores the need for flexible response mechanisms as frontline teams encounter real-world barriers [10, 14, 17, 18].

Despite this breadth, 30 ERIC strategies were absent from all included reviews, and many appeared to be underutilised. For example, no reviews described leveraging accreditation or regulatory levers. Financial incentives were almost entirely absent, with only two reviews mentioning dedicated funding and none describing modifications to reimbursement or bonus structures, despite repeated concerns about unfunded workload. In other clinical areas, even modest pay-for-performance payments have accelerated adoption of documentation and screening [49], yet PROM and PREM implementers appear to have left this mechanism largely unused. The absence of such strategies in the reviews may reflect broader funding constraints, which have been identified as a major barrier [26]. Future studies should explore the effectiveness of different financial incentives on PROM and PREM implementation.

Policy levers were similarly missing. We found no evidence that accreditation bodies, regulators, or executive directives were leveraged to make PROM and PREM capture a prerequisite for participation, even though national mandates exist for joint-replacement registries and cardiac surgery audits. Champions were common, but systematic mobilisation of high-influence opinion leaders, which typically outperforms informal championing, was not described, nor were narrative approaches such as video vignettes that translate abstract scores into compelling patient stories [50]. Dashboards were widespread, yet no review detailed the data architecture required to pool results across sites, stratify by equity variables and return insights in near real-time, an omission that could constrain scalability [51]. Similarly, patient-directed strategies were underused or narrowly applied, and were rarely supported by broader infrastructure such as language access services, public-facing outreach, or device provision [50].

Systems-level change often requires both additional resources and visible commitment from high-influence leaders, conditions that may be difficult to secure in smaller studies with constrained timelines, limited funding, and narrow implementation scopes. This contextual limitation likely contributes to the absence of policy-focused strategies in the reviewed literature. There is a critical need for future research and practice to explicitly integrate policy considerations, including exploring how regulatory incentives, accreditation requirements, and organisational mandates can be harnessed to drive adoption of PROMs and PREMs, standardise data practices, and ensure equitable participation. Developing this policy infrastructure through implementation research and practice is essential to bridge the gap between isolated pilot efforts and system-wide integration.

The lack of implementation strategies aimed at system-level change also has significant implications for equity. Few strategies explicitly sought to reduce disparities in PROM or PREM completion, interpretation, or follow-up. Additionally, although adaptation and tailoring to context, both important strategies for addressing health inequities [52], were mentioned; many important equity-focused implementation strategies, such as deploying community health navigators, offering linguistically and culturally adapted materials, providing device or connectivity support, and co-designing PROMs, PREMs, and implementation strategies with underserved patient groups were missing. This gap may reflect the nature of umbrella reviews: individual articles may have addressed equity without this being captured in the overall synthesis.

Our review translates findings into two tangible implementation planning tools that health system teams and researchers can use when implementing or evaluating PROMs and PREMs. First, the CFIR × ERIC matching matrix cross-walks 43 discrete implementation strategies against the 15 highest-evidence barriers, enabling implementers to quickly identify candidate strategies for specific barriers. Second, the EPIS-aligned roadmap sequences implementation strategies over time and specifies key deliverables. Practically, a health system team could use this roadmap to conduct a gap analysis of their current implementation plan, ensuring that strategies are in place across all four EPIS phases.

We did not assess the effectiveness of implementation strategies in enhancing the adoption, penetration, sustainability or scale-up of PROMs and PREMs. Future research is needed on several fronts to address this knowledge gap. First, evidence syntheses explicitly designed to pool effects, such as systematic reviews and meta-analyses of randomised hybrid or pure implementation trials, are needed to estimate the absolute and comparative impact of the individual strategies catalogued here. Second, new head-to-head, theory-driven cluster randomised trials should test single versus multicomponent implementation strategies in real-world services, with stratified analyses to explore context–mechanism–outcome interactions [50, 53–55]. At present, the field is dominated by hybrid type I and II designs that foreground clinical effectiveness while treating implementation as a secondary or co-primary aim [56–59]. Few trials are hybrid type III, and pure implementation trials remain rare [60–62]. This imbalance means we still lack causal evidence on which implementation strategy(ies) lead to the most durable gains in reach, fidelity, equity, and other implementation outcomes, and how those gains translate into patient outcomes [60].

### Strengths and limitations

This is the first study to use the ERIC taxonomy and the EPIS framework to identify, categorize and temporally map implementation strategies for PROMs and PREMs, offering phase-specific guidance. This secondary analysis has several strengths. The parent umbrella review followed the JBI guidance for umbrella reviews and drew on 25 reviews covering 1086 primary studies across nine clinical sectors, enhancing relevance beyond any single setting. All articles were independently double-coded using a recognized implementation strategy taxonomy, and data analysis was also conducted by two independent researchers. This study also has some limitations. Because we worked from published reviews, our synthesis is limited by the detail with which primary authors reported strategy dose, actors, and mechanisms, restricting depth of analysis. Temporal placement within EPIS phases rested on authors’ descriptions, so some misclassification is possible. Oncology and other high-income contexts dominate the evidence base, leaving paediatrics, long-term care, and low- and middle-income settings under-represented. Finally, the review maps what has been attempted rather than estimating effectiveness, so it can guide strategy selection but cannot yet rank strategies by impact.

## Conclusion

This study underscores that current PROM and PREM implementation efforts integration hinge on orchestrating people, processes and technology in tandem: early-phase engagement of clinicians, managers and patient partners must be paired with deliberate choices about measure design, accessible multilingual platforms for survey completion, and seamless IT architecture that populates dashboards and EHR alerts without added burden. We identified 43 implementation strategies, mapped them to the highest-priority CFIR barriers, and sequenced them across the EPIS timeline, offering two practical implementation planning aids: an action-oriented matrix and a phase-specific roadmap. Yet the evidence base still skews toward well-resourced oncology settings, leans on goodwill rather than durable financing or policy levers, and rarely reports equity safeguards. Future hybrid and implementation trials should therefore evaluate implementation strategies across a wider range of settings, including low-resource regions, so that patient-centred measurement becomes routine, resilient, and equitable at scale.

## Data Availability

All data generated during this study is included in the article and the appendix.
